# Production of a mono-biotinylated EGFR nanobody in the *E. coli* periplasm using the pET22b vector

**DOI:** 10.1186/s13104-018-3852-1

**Published:** 2018-10-22

**Authors:** Alfiah Noor, Gudrun Walser, Matthijs Wesseling, Philippe Giron, Albert-Menno Laffra, Fatima Haddouchi, Jacques De Grève, Peter Kronenberger

**Affiliations:** 1 Laboratory of Medical and Molecular Oncology, Vrije Universiteit Brussel, Laarbeeklaan 109, 1090 Brussels, Belgium; 2 Laboratory of Biotechnology, Erasmushogeschool Brussel, Laarbeeklaan 121, 1090 Brussels, Belgium

**Keywords:** Nanocarriers, EGFR, Nanobody, Single-domain antibody, VHH domain, Mono-biotinylation, Streptavidin, Cancer

## Abstract

**Objective:**

Our aim was to produce a mono-biotinylated single domain antibody (‘nanobody’) specific for the epidermal growth factor receptor (EGFR), which is overexpressed in many cancer cells. The binding of the nanobody and its function are tested in cancer cells. The construct could be used to carry variable therapeutic or diagnostic load using biotin-streptavidin bridging.

**Results:**

The EGFR-specific 7D12 nanobody was genetically fused to an IgA hinge linker and to a C-terminal biotin ligase acceptor sequence, allowing mono-biotinylation in *E. coli*. Expression was in strain BL21-DE3 from a T7 RNA polymerase driven pET22b vector. The biotinylated nanobody, isolated from the periplasm, was purified using streptavidin-mutein affinity chromatography. Final yields were up to 5 mg/l of cell culture. We showed that the construct could bind to EGFR expressing A431 epidermoid carcinoma cells, and to transiently transformed EGFR overexpressing HEK293T cells and not to EGFR negative control cells. The specificity for the EGFR was further demonstrated by immunoprecipitation. To test the functionality, PC9 non-small cell lung cancer cells were treated with mono-biotinylated nanobody or with streptavidin-coupled tetravalent nanobodies. Both were able to block mutant EGFR phosphorylation and slow down growth of PC9 cells. Tetravalent nanobodies were able to downregulate AKT phosphorylation.

**Electronic supplementary material:**

The online version of this article (10.1186/s13104-018-3852-1) contains supplementary material, which is available to authorized users.

## Introduction

Nanocarriers for nanomedicine purpose are molecular complexes at a 100 nm scale that carry a therapeutic cargo [1]. The construction of targeted nanoparticles implies that the construct can be taken up by specific cell populations. The use of antibodies has been an evident choice to construct such targeted particles [2]. However, antibodies are expensive to produce and difficult to assemble in recombinant form. Their relatively big size of about 150 kDa/~ 30 nm, hinders uptake and/or penetration in the cell [2]. Hamers et al. discovered that IgG antibodies from camelid species are composed of heavy chains only [3]. The single peptide, antigen-binding domains of these heavy chains can be produced as functional fragments, known as nanobodies (developed by Ablynx, Belgium) [4]. Nanobodies (~ 15 kDa) can be produced in *E. coli* and are very soluble and stable in various conditions [5–7]. The nanobodies contain a single disulfide bridge, and in *E. coli* they are therefore preferentially isolated from the oxidizing environment of the periplasm.

Streptavidin is a ~ 56 kDa homo-tetramer that binds selectivity with four biotin molecules [8]. The streptavidin–biotin interaction is widely used in molecular science and has been developed for assembling nanoparticles. Previously Hernot et al. described the production of mono-biotinylated green fluorescent protein (eGFP) and vascular cell adhesion molecule-1 (VCAM-1) nanobodies [9]. These nanobodies were subsequently coupled to biotinylated lipid to assemble targeted microbubbles for use in ultrasound imaging of tumors.

The epidermal growth factor receptor (EGFR) is mutated or overexpressed in several human epithelial cancers. The EGFR is a validated therapeutic target in various cancers, employing monoclonal antibodies and tyrosine kinase inhibitors [10, 11]. Nanobodies against the EGFR were developed by some research groups [12, 13]. Roovers et al. used biotinylated EGFR nanobodies for the purpose of EGFR immunoprecipitation [12], but the sequence of the construct was not specified. A mono-biotinylated nanobody could be used to direct various therapeutic agents to EGFR overexpressing cancer cells.

## Main text

### Methods

#### Production of EL2BH nanobody

**Fig. 1:**
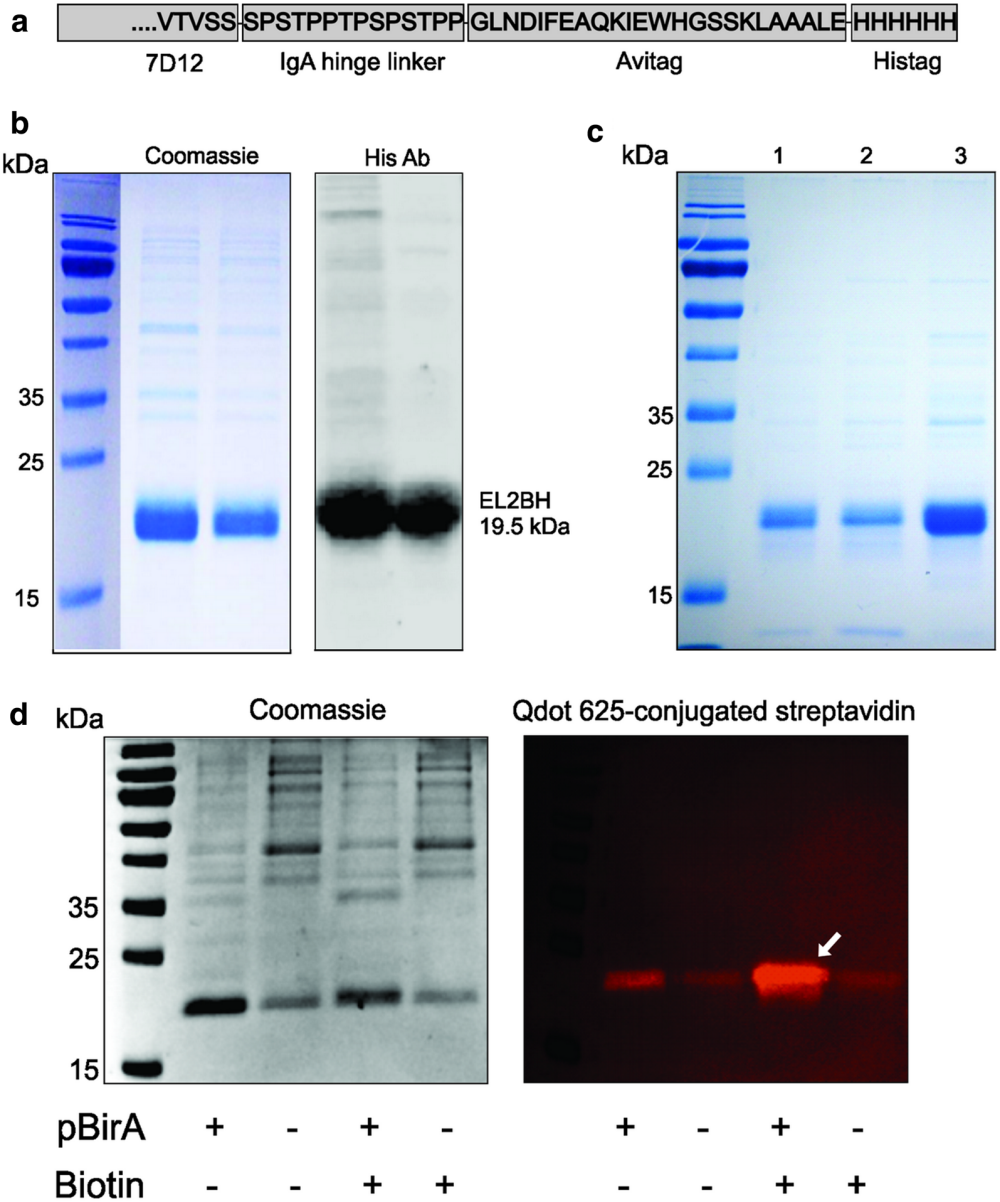
EL2BH isolation and purification by streptavidin-mutein. **a** C-terminal sequence of EL2BH. **b** EL2BH from two preparations analyzed in SDS-PAGE and stained with Coomassie blue (left panel) or transferred to a PVDF membrane and further stained with His tag antibody (right panel). **c** SDS-PAGE of biotinylated nanobody EL2BH affinity purified by streptavidin-mutein using either 0.5 M (lane 1), or 1 M ammonium sulphate (lane 2). At 1 M ammonium sulphate much of the nanobody sticks to the column matrix and can be eluted using biotin and mechanical resuspension of the matrix (lane 3). Upon elution with biotin at 0.5 M ammonium sulphate, no EL2BH remained on the matrix (not shown). **d** SDS PAGE and western blot from crude periplasmic extracts obtained from 1 mmol/1 IPTG (Promega) induced cultures for 1 h at 36 °C that express both EL2BH and BirA, or EL2BH only. Both strains were subsequently grown in LB medium containing either 50 µM biotin, or no extra biotin. Following IPTG induction of all cultures, and incubation for 1 h at 36 °C, periplasmic extracts were analyzed by SDS-PAGE (left panel), and blotted proteins were visualized by QDot-625-conjugated streptavidin (ThermoFisher) under ultraviolet light (geldocEZ, Biorad) (right panel). The arrow points at the highest biotinylation signal that is obtained by overexpression of BirA and addition of extra biotin in the medium

The sequence of EGFR nanobody 7D12 was reverse translated (Emboss backtranseq) with codon usage of *E. coli* K12. An “avitag” biotin acceptor sequence GLNDIFEAQKIEWH (Avidity) was added to the C-terminal end, separated from the nanobody sequence by a Llama linker, i.e. an immunoglobulin IgA hinge STPPTPSPSTPP [9]. The resulting sequence ends with a vector-derived hexa-histidine tag at the C-terminus (Fig. 1a, sequence in Additional file 1). The mono-biotinylated 7D12 EGFR nanobody construct will be referred to as EL2BH.

The EL2BH synthetic DNA sequence was ordered from GenScript and subcloned into vector pET22b (EMDMillipore) between *Eco*RI and *Hin*dIII sites. The EL2BH nanobody was isolated from the periplasmic space of *E. coli* BL21-DE3 (EMDMillipore) that we previously transformed with plasmid pBirACm (Avidity) encoding an IPTG inducible biotin ligase. The periplasmic extract was prepared essentially according to the pET system manual (TB055 rev C 11th edition, Novagen/EMDMillipore) and the sterile filtered extract in 5 mM MgSO_4_ was affinity purified using streptavidin-mutein gel (Roche) according to manufacturer’s instructions. The eluate was concentrated by ultrafiltration (Amicon, MWCO 10 kDa), and free biotin was removed by gel filtration (Zeba spin columns, MWCO 7 kDa, ThermoFisher). The concentration of EL2BH was estimated on a Shimadzu biospec-nano, using a molar mass of 19,531 Da and a molar extinction coefficient of 39,545/M/cm.

#### Immunoprecipitation

Streptavidin agarose (ThermoFisher) was preloaded with 10 µg EL2BH nanobody or 1 µg poly-biotinylated EGFR antibody control (Abcam) and incubated at 4 °C overnight, followed by the addition of A431 cell lysate and further incubation for 1 h at 4 °C. The mixture was washed extensively and then denatured with SDS-PAGE loading buffer at 95 °C. Following SDS-PAGE and transfer to a PVDF membrane, detection was using an anti-EGFR antibody (Sigma), an HRP-conjugated anti-mouse IgG (GE Healthcare) and chemiluminescence detection (Advansta) using the LI-COR Odyssey^®^ Fc imaging system.

#### Cell transfections

HEK293T cells at over 70% confluence were transfected with EGFR pCDNA plasmid using Lipofectamine 2000 according to manufacturer’s instructions (ThermoFisher).

#### Western blot

Cells were lysed in 1% Triton-X100, Tris/HCl 20 mM, NaCl 150 mM, EDTA 1 mM, Na–pyrophosphate 2.5 mM, sodium orthovanadate (Na_3_VO_4_) 1 mM, leupeptin 1 µg/ml, protease inhibitors 1% and phosphatase inhibitors 1% (Sigma). Protein concentration was measured by the Bradford assay (Bio-Rad). Following SDS-PAGE, samples were blotted to PVDF membranes and probed with QDot-625-conjugated streptavidin (ThermoFisher) or with indicated primary antibodies: pEGFR (Tyr1068) and pERK (Tyr185/187) (Cell signaling); pAKT (Ser473) (ThermoFisher); His Tag (Genscript); and β-actin (Sigma). Detection of QDot 625-conjugated streptavidin was done under ultraviolet light (geldocEZ, Bio-Rad). Primary antibodies were bound to HRP-conjugated secondary antibodies (Anti-mouse/anti-rabbit, GE Healthcare) and visualized by chemiluminescence (Advansta) with a LI-COR Odyssey^®^ Fc imaging system.

#### Immunofluorescence assay

Mono-biotinylated EL2BH nanobody (5 µg) or poly-biotinylated EGFR antibody (1 µg) as control were pre-mixed with streptavidin Alexa fluor 488 (ThermoFisher) and incubated at 4 °C for 1 h. The complex was added to EGFR transfected or non-transfected HEK293T cells in PBS and further incubated at 37 °C for at least 1 h. The cells were washed and stained with Hoechst 3342 (ThermoFisher) for nuclear counterstaining. Images were captured using an EVOS fluorescence microscope.

#### Flow cytometry analysis

A431 cells were trypsinized (Roth) and resuspended in ice cold PBS contain 10% newborn calf serum (Sigma) after which 2 µg poly-biotinylated antibody or 2 µg EL2BH nanobody was added. Incubated was for 1 h at 4 °C. The cells were washed 3× with PBS containing 3% biotin-free BSA (Roth). Streptavidin-Alexa Fluor 488 (2 mg/ml, ThermoFisher) was diluted 1:100 in PBS with 3% biotin-free BSA and incubated for 30 min in the dark at 4 °C. The cells were washed 3× and resuspended with PBS containing 3% biotin-free BSA and analyzed on a Beckman Coulter Epics XL-MCL flow cytometer.

#### Cell confluency analysis

PC9 cells (Sigma) were grown in RPMI-1640 (ThermoFisher) 24 h prior to the addition of nanobody, streptavidin or the combination of both. Cells were monitored every hour in the IncuCyte^®^ live-cell analysis system and cell confluency was analyzed with IncuCyte^®^ ZOOM software.

### Results and discussion

#### Isolation and purification of EL2BH nanobody

The use of mono-biotinylated nanobodies for the construction of nanoparticles has been described by Hernot et al. [9]. However, the yield of both biotinylated-nanobodies was low (0.21–0.26 mg/l), and this may hamper the routine use of the biotin-streptavidin technology. We sought to increase the yield by using pET expression vectors that are well known for their fast production and high yield [14, 15], and by optimizing the isolation protocol. Using this approach, the yield of mono-biotinylated EGFR nanobody could be increased to 5 mg/l.

Nanobodies contain a single immunoglobulin disulfide bridge and are therefore favorably expressed in the oxidizing environment of the periplasm [14]. In this study, the periplasmic extract contains protein with the expected size of 19.5 kDa (Fig. 1b, left panel), and the identity of EL2BH was further confirmed by hexa-histidine immunostaining (Fig. 1b, right panel).

The production of milligrams amounts of mono-biotinylated nanobody was found to correlate with the sequence of the N-terminus. When cloned between *Eco*RI and *Hin*dIII, the construct starts with the pET22b’s pelB leader MKYLLPTAAAGLLLLAAQPAMA, followed by vector derived sequence MDIGINSDPNSMA down to the *Eco*RI site. This short vector derived sequence is part of the pET22b cloning site and has no biological origin or correlate. When cloned between *Nco*I and *Hin*dIII, the construct’s N-terminus contains the pelB leader, immediately followed by the nanobody’s methionine. It was found that this construct produced a much lower yield (data not shown), such that it’s further use was abandoned. One possibility is that the 13 vector-derived amino acids positively affect the efficiency of transit by the pelB leader into the periplasm, and thereby increase the yield.

The yield was also increased when the cultures were induced with IPTG, not at A_600_ 0.5–1.0 as described in many protocols, but in the later log phase, i.e. A_600_ = 1.3. Growth was continued for 1 h 30 with vigorous shaking at 200 rpm. It was found that induction temperatures between 33 and 37 °C did not have a significant effect on the yield of periplasmic nanobody construct (data not shown). A routine induction temperature was set to 36 °C.

We also found that for efficient binding to the mutein, a minimum concentration of 500 mM ammonium sulphate was required. 1 M ammonium sulphate increased the yield of bound nanobody significantly, but resuspension of the matrix in 2 mM biotin was needed for elution. The high concentration of ammonium sulphate probably increased the hydrophobicity of the nanobodies (Fig. 1c, lane 3).

To obtains information on the effectiveness of the in vivo biotinylation, the EL2BH-pET22b construct was transformed into *E. coli* BL21-DE3 hosts containing either the biotin ligase encoding plasmid pBirACm, or without plasmid. The samples were analyzed using SDS PAGE (Fig. 1d, left panel) and biotinylation was detected using fluorescent QDot-625 coupled streptavidin (Fig. 1d, right panel). The highest biotinylating signal (arrow) was obtained when both the biotin ligase is overexpressed, and extra biotin is present in the medium (Fig. 1d, lane 3, right panel). The background signals in the other cultures can be explained by the presence of biotin in the LB medium, and by the activity of the natural biotin ligase from *E. coli*.

#### Characterization of EL2BH nanobody on EGFR expressing cells

**Fig. 2:**
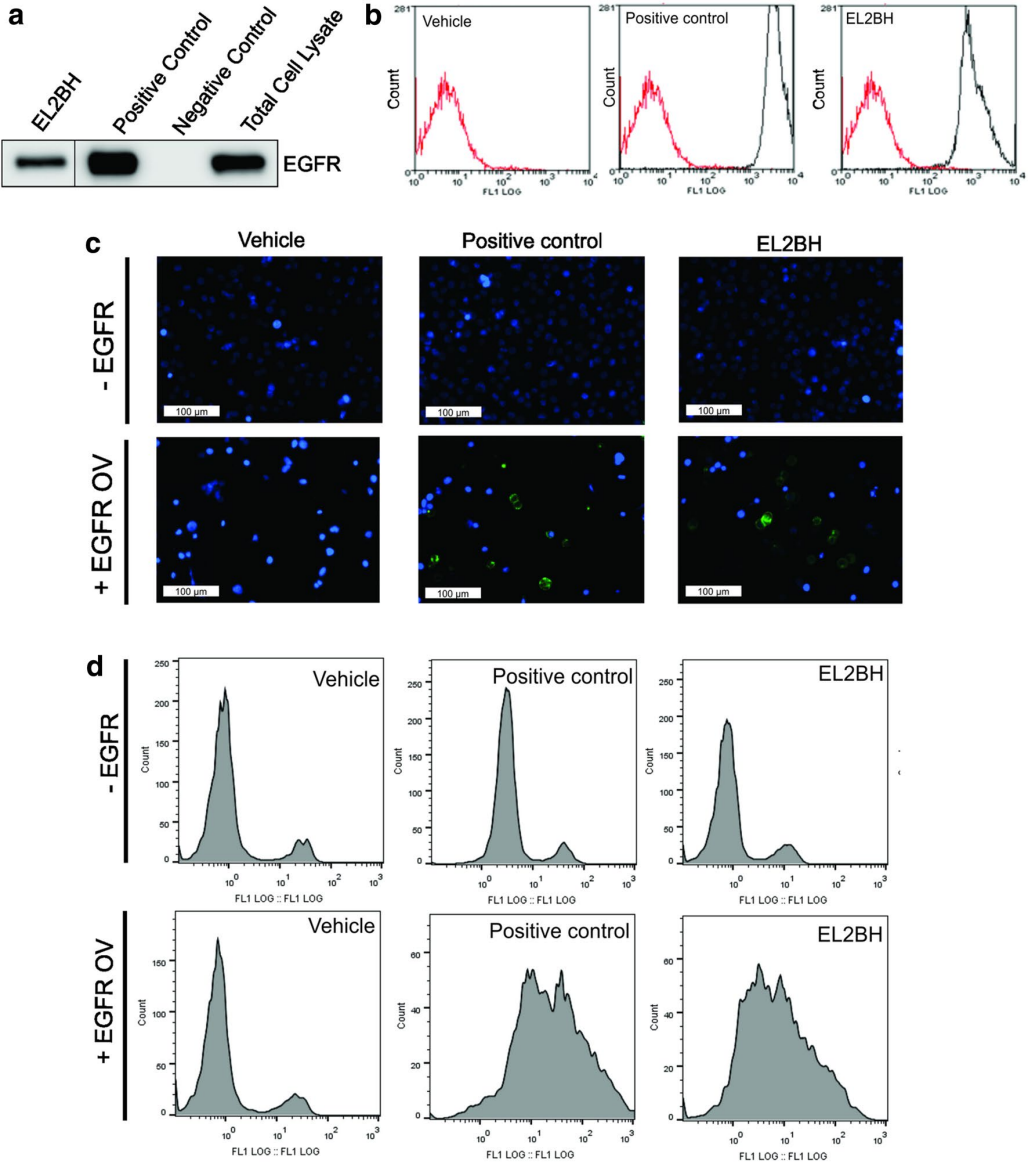
Mono-biotinylated EL2BH binds to EGFR in EGFR expressing cells. **a** Immuno-precipitation and western blotting of EGFR from A431 cell lysates using EL2BH nanobody, or commercial poly-biotinylated EGFR antibody as positive control, both pre-conjugated to streptavidin agarose beads. Streptavidin agarose beads in PBS with or without antibody were used as a negative control. **b** Flow cytometry analysis of A431 cells stained with EL2BH nanobody pre-coupled to streptavidin-Alexa Fluor 488, compared to positive control antibody pre-coupled to streptavidin-Alexa Fluor 488 and vehicle control with streptavidin-alexa 488 only. **c** Immunostaining of EGFR-negative and EGFR-overexpressing HEK293T cells (EGFR OV) with EL2BH pre-coupled to Streptavidin Alexa Fluor 488 (green), counterstained with Hoechst 3342 (blue). **d** Flow cytometry of EGFR-negative and EGFR-overexpressing HEK293T cells (EGFR OV) with EL2BH pre-coupled to streptavidin-Alexa Fluor 488. The flow cytometry data were analyzed using Flowjo software

The binding capacity of 7D12 was first tested in A431 cells because the 7D12 nanobody was constructed by immunizing a Llama with EGFR overexpressing A431 epidermoid carcinoma cells [12]. A431 cell lysates were incubated with EL2BH and immunoprecipitated using streptavidin coupled agarose beads. The results in Fig. 2a demonstrated that ELBH nanobodies were able to bind to the EGFR in A431 cells. The flow cytometry results indeed showed that EL2BH can be detected at the surface of A431 cells (Fig. 2b).

**Fig. 3:**
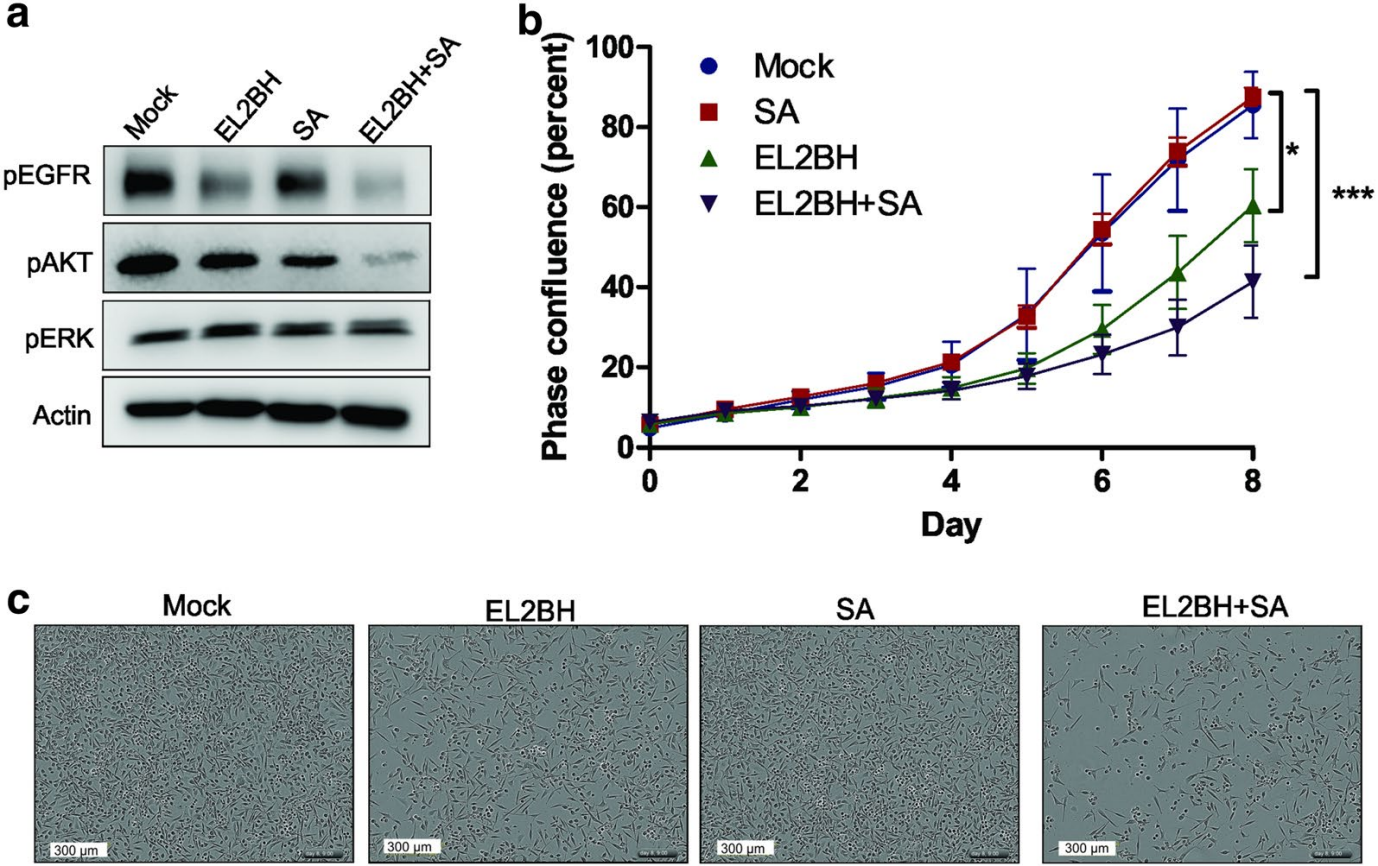
Effect of monovalent or tetravalent EL2BH on PC9 cells. **a** After inoculation of PC9 cells with monovalent EL2BH nanobody, streptavidin (SA), tetravalent EL2BH (EL2BH + SA) or PBS as vehicle control, cells were incubated for 24 h in low serum condition (Opti-MEM), and cell lysates were prepared. Proteins were size-separated by SDS-PAGE and blotted onto PVDF membranes. Membranes were then stained with antibodies against phosphorylated EGFR (Tyr1068), pAKT (Ser473), pERK (Tyr185/187), and for actin as loading control. **b** PC9 cells were seeded in a 96 well plate 24 h prior to inoculation as in **a** and cell confluency was further observed in an IncuCyte^®^ live cell analyzer for 8 days. The differences between groups were calculated by comparing the area under the curve (AUC) of each phase confluency graph from 4 different experiments, using ANOVA followed by the Tukey test. The difference between groups was considered statistically significant when *P* < 0.05*, *P* < 0.01** and *P* < 0.001***. **c** Representative images from **b** on day 8

To confirm the binding of EL2BH nanobody to EGFR, HEK293T cells were transfected with an EGFR-encoding plasmid to obtain EGFR overexpressing cells. EL2BH nanobody and the positive control antibody were pre-incubated with streptavidin-Alexa Fluor 488 and the complexes were incubated with HEK293T EGFR-negative control cells or EGFR-overexpressing cells. We observed positive stained cells (green) upon incubation with positive control and EL2BH nanobody (Fig. 3c). Flow cytometry further confirmed that EL2BH only binds to EGFR-overexpressing cells (Fig. 3d).

Our results thus show that fusing several sequences (a short N-terminal vector sequence, the 7D12 sequence, a Llama linker, the biotin acceptor and a hexa-histidine tag) did not interfere with the binding of the EL2BH nanobody to the EGFR.

#### The effect of EL2BH nanobody-streptavidin complex in PC9 cells

To verify the therapeutic properties of the nanobody, EL2BH was studied on PC9 non-small cell lung cancer cells. These cells harbor an EGFR-activating deletion in exon 19 and have a modest EGFR gene amplification. Cells were treated with monovalent or tetravalent EL2BH (pre-bound to streptavidin). EGFR activity and the signaling pathway were analyzed by western blot. The result shows that EGFR phosphorylation was inhibited after treatment with EL2BH, both by the monovalent or the tetravalent EL2BH (Fig. 3a). This result supports previous findings with the 7D12 EGFR nanobody, in which the nanobody could block ligand binding and further block EGFR activity [12, 16]. In addition, we find that EL2BH can also block mutant, activated EGFR function which is also ligand independent. Interestingly, tetravalent EL2BH downregulated AKT phosphorylation, but the monovalent EL2BH did not. We postulate that tetravalent EL2BH but not monovalent EL2BH can interfere with EGFR dimerization and even with HER3 heterodimerization, which would effectively inhibit AKT activation [17]. PC9 cells have a constitutively activated EGFR, which may explain why ERK activity was not affected by either treatment.

Next, the biological activity of EL2BH on PC9 cells was further analyzed by monitoring cell confluency. In Fig. 3b, streptavidin-bound, tetravalent EL2BH decreased cell growth more than monovalent EL2BH. The control with streptavidin only had no effect. These results therefore are in line with the finding by Roovers et al. that a nanobody in bivalent form is more potent compared to the single form [18].

Mono-biotinylated nanobodies have a broad application, since streptavidin bridging allows for the coupling of any biotinylated molecule, or streptavidin conjugate, many of which are commercially available. The EL2BH nanobody could be used for nanoparticle construction and delivery of a diagnostic or therapeutic cargo to EGFR overexpressing cancer cells.

## Limitations

- That the EL2BH nanobody is functional does not imply that assemblies using EL2BH are functional as well. Therefore, further experiments must be done to attach EL2BH to nanoloads via biotin-streptavidin bridging.
- The use of streptavidin, isolated from Streptomyces, may yield immunogenicity issues when used in nanoparticle applications in vivo. It may be necessary to use mutant streptavidin with reduced immunogenicity.

## Additional file

**Additional file 1.** EL2B *Eco*RI–*Hin*dIII insert in pET22b plasmid.

## Abbreviations

BSA: bovine serum albumin
EGFR: epidermal growth factor receptor
EL2BH: EGFR + Linker nr. 2 i.e. Llama IgA hinge + Biotin avitag + Hexa histidine tag
HRP: horseradish peroxidase
IPTG: isopropyl β-d-1-thiogalactopyranoside
PVDF: polyvinylidenefluoride
SDS-PAGE: sodium dodecyl sulphate–polyacrylamide gel-electrophoresis
