# Supplementary material for: Production of a mono-biotinylated EGFR nanobody in the E. coli periplasm using the pET22b vector
Source: BMC Res Notes. 2018 Oct 22;11:751. doi: 10.1186/s13104-018-3852-1 (PMC6196415; doi:10.1186/s13104-018-3852-1)
Supplement: Supplementary file 1 — Additional file 1. EL2B EcoRI–HindIII insert in pET22b plasmid. [file 13104_2018_3852_MOESM1_ESM.docx]

**Additional Data**

EL2B insert EcoRI-HindIII in pET22b:

Start with: GAATTC

| 1  61  121  181  241  301  361  421 | AATGGCGCAG GTGAAACTGG AAGAAAGCGG CGGCGGCAGC GTGCAGACCG GCGGCAGCCT  GCGCCTGACC TGCGCGGCGA GCGGTCGCAC CAGCCGCAGC TATGGCATGG GCTGGTTTCG  CCAGGCGCCG GGCAAAGAAC GCGAATTTGT GAGCGGCATT AGCTGGCGCG GCGATAGCAC  CGGCTATGCG GATAGCGTGA AAGGCCGCTT TACCATTAGC CGCGATAACG CGAAAAACAC  CGTGGATCTG CAGATGAACA GCCTGAAACC GGAAGATACC GCGATTTATT ATTGCGCAGC  AGCAGCAGGT AGCGCGTGGT ATGGCACCCT GTATGAATAT GATTATTGGG GCCAGGGCAC  CCAGGTGACC GTGAGCAGCA GCCCGAGCAC CCCGCCGACC CCGAGCCCGA GCACCCCGCC  GGGCCTGAAC GATATTTTTG AAGCGCAGAA AATTGAATGG CATGGCAGCA GC |
| --- | --- |

End with: AAGCTT
